# Acute Gastric Volvulus Causing Splenic Avulsion and Hemoperitoneum

**DOI:** 10.1155/2018/2961063

**Published:** 2018-04-01

**Authors:** Yana Cavanagh, Neal Carlin, Ruhin Yuridullah, Sohail Shaikh

**Affiliations:** ^1^Department of Gastroenterology, New York Medical College at St. Joseph's Regional Medical Center, Paterson, NJ, USA; ^2^Department of Medicine, New York Medical College at St. Joseph's Regional Medical Center, Paterson, NJ, USA

## Abstract

Gastric volvulus is an abnormal, potentially life-threatening, torsion of the stomach. The presence of complications such as hemoperitoneum increases the diagnostic urgency; however it can also mask the presentation of gastric volvulus. We encountered a 66-year-old female who presented with symptomatic gastric outlet obstruction and was found to have hemoperitoneum and splenic avulsion on imaging. In our case, hemoperitoneum was a clinical red herring as initial imaging concentrated on the presence of hemoperitoneum and was nondiagnostic of gastric volvulus. Interestingly, our patient experienced complete resolution of her presenting symptomatology following placement of a nasogastric tube. Furthermore, endoscopic evaluation revealed no overt pathology to explain outlet obstruction. In light of these findings, gastric torsion was strongly suspected. A repeat CT scan was confirmatory, elucidated reduction of the stomach to its anatomic position, retroactively diagnosing a gastric volvulus. This case is unusual in its presentation and setting. The patient presented with two rare complications of gastric volvulus, hemoperitoneum and splenic avulsion. Additionally, ten years prior to this presentation the patient had a temporary gastrostomy tube. Gastropexy with a gastrostomy is the treatment for gastric volvulus and should have been preventative of her presentation with torsion. Furthermore, the gastric volvulus was not initially recognized radiographically due to the presence of masking radiographic findings. This case serves to highlight the utility of clinical acumen and maintain a high index of suspicion for gastric volvulus in all cases presenting with Borchardt's triad.

## 1. Introduction

Gastric volvulus is an abnormal rotation of the stomach that is well documented in small animals [1–4]. However, gastric volvulus is a rare entity in humans [5–7]. The peak incidence of gastric volvulus is in the fifth decade. No association with race or sex has been established; however gastric volvulus is associated with significant mortality [7, 8]. Therefore, prompt diagnosis and treatment are imperative [9, 10].

Acute gastric volvulus typically presents with pain in the upper abdomen or lower chest and is associated with vomiting. These symptoms accompanied by an inability to pass a nasogastric tube are known as Borchardt's triad, which is characteristic of gastric volvulus [7, 11]. The main consequence of the gastric volvulus is acute, intermittent, or chronic foregut obstruction [8, 12, 13]. Associated complications can include ulceration, perforation, hemorrhage, pancreatic necrosis, and omental avulsion [14–16]. On rare occasions, rotation of the stomach causing disruption of the splenic vessels or splenic rupture has been reported [9, 17].

**Figure 1:**
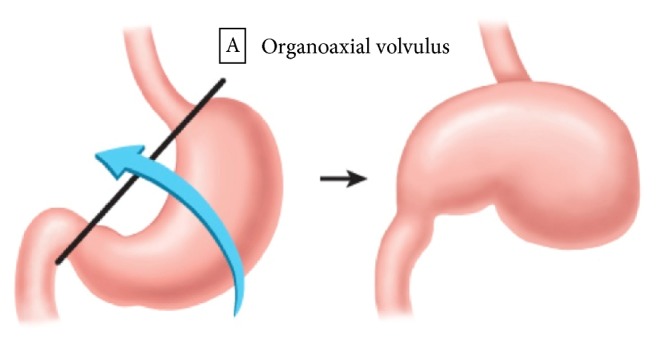
*Organoaxial gastric volvulus.* Rotation of the stomach along its long axis through a line that connects the gastroesophageal junction and the pylorus. The antrum rotates anterosuperiorly and the fundus rotates posteroinferiorly. The greater curvature of the stomach comes to rest superior to the lesser curvature of the stomach in an inverted position.

The most frequently implemented classification of gastric volvulus is based on the axis around which the stomach rotates [18, 19]. Organoaxial gastric volvulus (Figure 1) is rotation of the stomach around its long axis. Organoaxial volvulus is the most common type, occurring in sixty percent of cases [7, 19]. It is associated with secondary etiologies such as a laxity of gastric ligaments or diaphragmatic defects [8, 19–21]. Strangulation and necrosis can occur with this type of volvulus [7].

**Figure 2:**
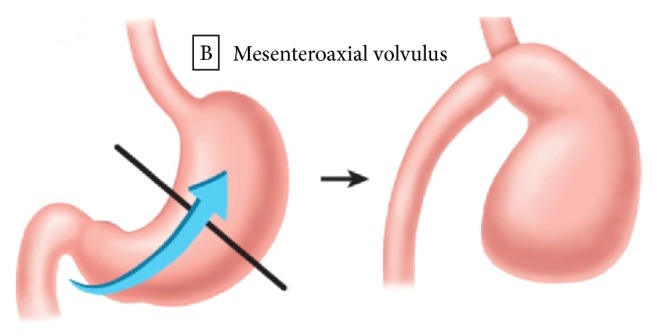
*Mesenteroaxial gastric volvulus.* The stomach rotates around its short axis through a perpendicular line connecting the greater and lesser curvatures of the stomach. The antrum becomes displaced above the gastroesophageal junction.

Rotation of the stomach along its short axis or mesenteroaxial volvulus causes the antrum to become displaced above the gastroesophageal junction (Figure 2). This form of volvulus is usually partial (<180°), not generally associated with a secondary anatomic defect, and vascular compromise is uncommon [19]. A combined gastric volvulus, in which the stomach twists mesentericoaxially and organoaxially, is rare but can be seen in patients with chronic volvulus [18, 22]. Gastric volvulus can also be described as idiopathic (type I) or acquired (type II) [7, 23].

## 2. Case Report

We encountered an unusual case of organoaxial gastric volvulus resulting in splenic laceration and hemoperitoneum in a patient with a distant history of gastrostomy tube. A 66-year-old Hispanic female with a history of stroke and residual expressive aphasia as well as right sided hemiparesis presented with acute onset abdominal pain of one-day duration. The pain was described as 7/10, achy, and diffuse. It was associated with multiple episodes of nonbloody emesis of food contents and white frothy fluid. There were no alleviating or aggravating factors noted and she had never experienced similar symptoms in the past. She denied preceding trauma, chest pain, change in bowel habits, consumption of new/unusual foods, or sick contacts.

**Figure 3:**
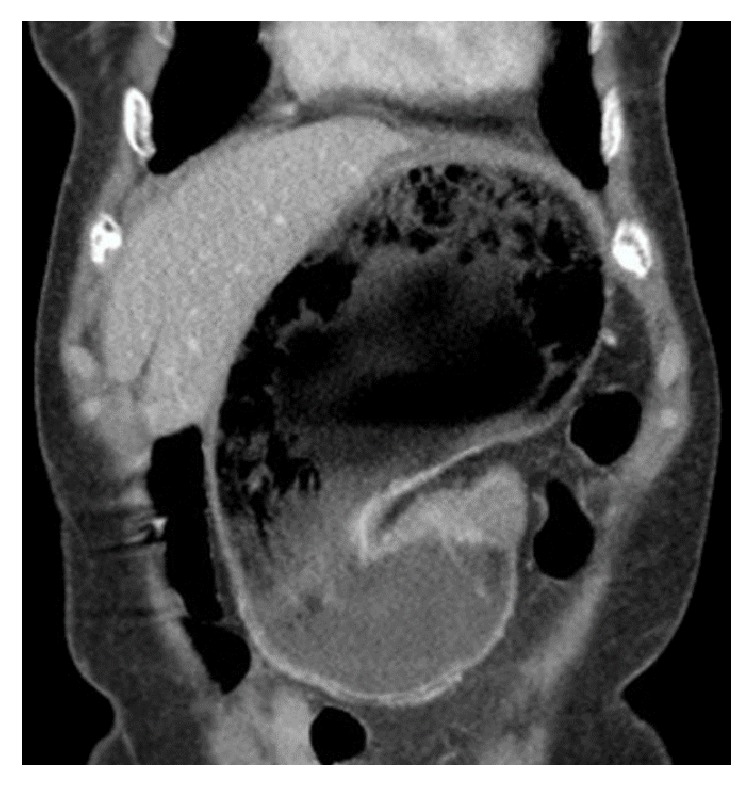
CT of abdomen and pelvis with contrast showing a markedly distended abdomen without focal wall thickening. The J-shaped stomach is rotated along its long axis, displaying a gastric volvulus. Fluid, consistent with blood, is seen tracking along the paracolic gutters and the pelvis.

On physical examination, the patient appeared uncomfortable and had a well-healed cicatrix from previous gastrostomy tube in the left upper quadrant. Her examination revealed abdominal distention as well as moderate tenderness to light palpation, diffusely. Pertinent negatives included the absence of guarding, rigidity, or rebound tenderness. Her laboratory evaluation revealed an acute normocytic anemia with a hemoglobin of 11.9 and hematocrit of 36.9. A complete metabolic panel was unremarkable and her lactic acid was 1.1. An emergent CT scan of the abdomen and pelvis reported a grossly distended stomach with gastric outlet obstruction, as well as a moderate amount of hyperdense intraperitoneal fluid, highly concerning for hemoperitoneum (Figure 3).

**Figure 4:**
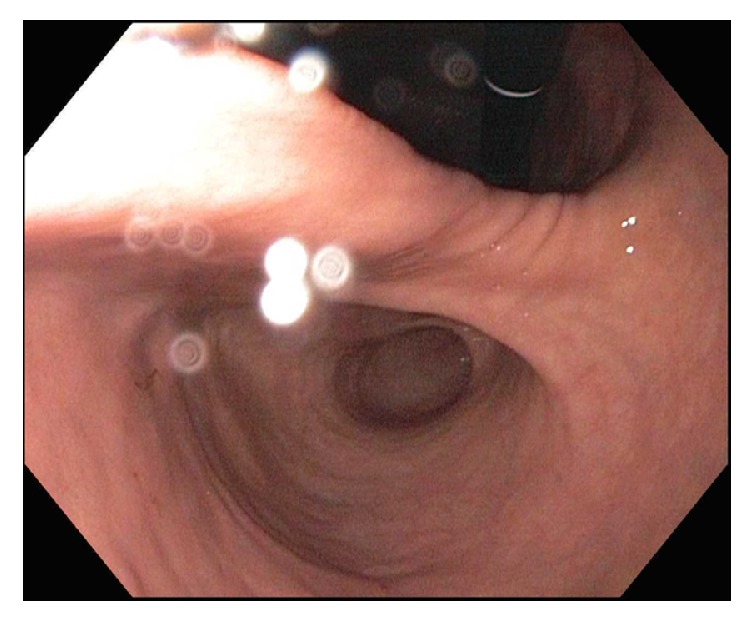
Retroflexed endoscopic view of the lesser curvature of the body of the stomach, antrum, and fundus, depicting the deeply J-shape anatomy of the stomach.

**Figure 5:**
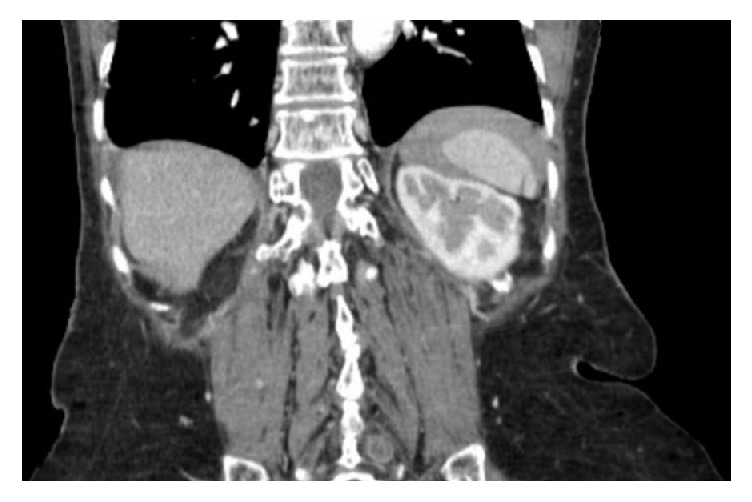
Repeat CT of abdomen and pelvis with contrast revealing an apparent linear defect measuring 0.9 cm involving the posteroinferior margin of the spleen. Again seen, consistent with prior study is the presence of a moderate amount of hyperdense free intraperitoneal fluid, indicative of hemoperitoneum.

While awaiting abdominal CT findings, a nasogastric tube (NGT) was placed for symptomatic care. One and a half liters of nonbloody, nonbilious gastric fluid was aspirated with concomitant resolution of her presenting complaints upon decompression of her stomach. Gastroscopy was performed to further elucidate the etiology of the patient's gastric outlet obstruction. Endoscopy revealed distal Los Angeles Grade C esophagitis, a deeply J-shaped stomach, a 10 mm gastric body ulcer with pigmented spots, and nonbleeding erosive gastropathy in the antrum. The duodenum was easily accessible and unremarkable (Figure 4). Following gastroscopy and further review of the initial imaging, a reduced gastric volvulus was strongly suspected and a repeat CT scan was pursued. It revealed the stomach in an anatomically differing position from initial imaging, confirming the presumptive diagnosis, as well as persistent stable hemoperitoneum and a 0.9 cm linear defect involving the posteroinferior margin of the spleen (Figure 5).

As the volvulus resolved with medical management, no surgical interventions were pursued. The potential for recurrence of the volvulus was discussed with the patient and her family and symptomatic care was preferred. The NGT remained in place for one day while the patient's diet was advanced to clear liquids and then to regular diet. The patient tolerated diet and was discharged home in stable condition.

## 3. Discussion

Gastric volvulus is an abnormal rotation of the stomach along its either long or short axis. Prompt diagnosis and treatment are imperative in order to avoid potential complications such as ulceration, perforation, hemorrhage, pancreatic necrosis, and omental avulsion. In our case, the patient presented with acute onset abdominal pain associated with multiple episodes of emesis. CT scan revealed a distended, deeply J-shaped stomach, gastric outlet obstruction, and moderate hemoperitoneum. Although no torsion was reported on initial imaging, a high level of suspicion was maintained for gastric volvulus due to the congruency between the patient's presentation and Borchardt's triad. Gastroscopy displayed no overt etiology for gastric outlet obstruction, which was further supportive of a reduced volvulus, as placement of the NGT likely reduced the stomach to its anatomic position and therefore resulted in resolution of the patient's symptomatology.

**Figure 6:**
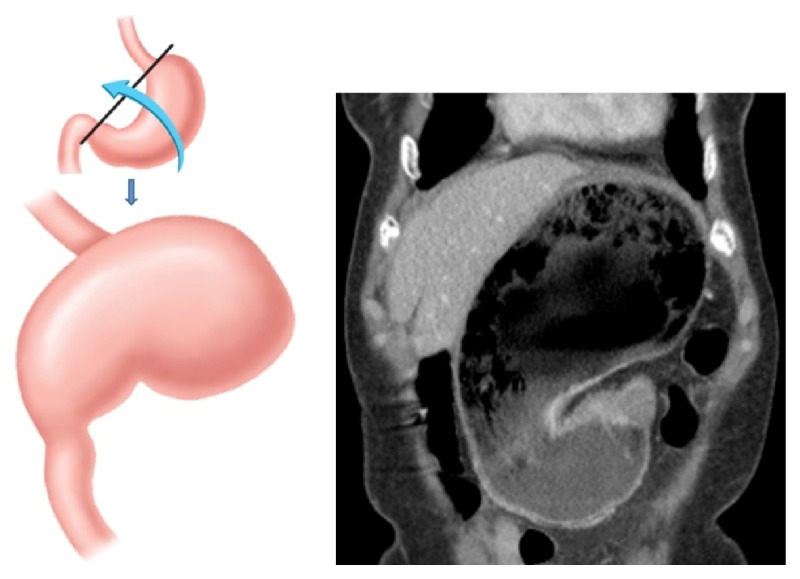
*Organoaxial gastric volvulus.* Rotation of the stomach along its long axis through a line that connects the gastroesophageal junction and the pylorus.

Repeat CT scan performed after endoscopy confirmed our supposition and showed the stomach was reduced to its normal anatomical position. Additionally, it revealed a 0.9 cm linear defect of the spleen, consistent with gastric volvulus causing splenic avulsion by placing traction on the splenogastric ligaments and resulting in hemoperitoneum. These radiographic findings and the patient's clinical course pointed to an initially overlooked radiographic finding of gastric volvulus due to the presence of red herring findings such as hemoperitoneum (Figure 6).

Our patient's unique presentation makes this case noteworthy. Gastric volvulus, particularly when accompanied by other complications, is rare. The treatment of gastric volvulus generally involves reduction of the stomach to its anatomic position and gastropexy by gastrostomy tube. This fixates the stomach to the abdominal wall, to prevent repeat torsion. Exceedingly interestingly, our patient previously had a gastrostomy tube following her cerebrovascular accident, which should have been preventative of future torsion. However, the deeply J-shaped anatomy of our patient's stomach nullified this additional point of fixation. The location of her previous PEG was the distal antrum, allowing ample unfixed proximal stomach to result in organoaxial rotation. As a result of this case, we urge maintaining a keen clinical eye and high level of suspicion for all cases presenting with Borchardt's triad, despite other potentially distracting radiographic and physical findings.
